# Blood Lead and IQ in Older Children

**DOI:** 10.1289/ehp.113-a581c

**Published:** 2005-09

**Authors:** David O. Carpenter

**Affiliations:** Institute for Health and the Environment University at Albany, Rensselaer, New York, E-mail: carpent@uamail.albany.edu

In their article about blood lead and IQ in older children, Chen et al. (2005) made the very important observation that IQ (intelligence quotient) in older children correlates better with their current blood lead level than with levels determined at 2 years of age. This observation has enormous public health implications in terms of defining who is at risk of cognitive decrement upon exposure to lead, and challenges the widely held assumption that the effects of lead on neurobehavioral function are exclusively developmental. My colleagues and I (Carpenter et al. 2002) previously studied the effects of gestational and lactational exposure of rats to lead with measurement of long-term potentiation in hippocampal brain slices. Long-term potentiation is an electrophysiological measure of synaptic plasticity that is widely viewed as being at least one component of learning and memory, and it is reduced upon *in vivo* lead exposure. To our surprise we also found that acute perfusion of low concentrations of lead onto brain slices from control animals resulted in reduction of long-term potentiation, suggesting that the effect is more pharmacologic than developmental. The results of Chen et al. (2005) in humans and our studies in rats suggest that lead causes both developmental and pharmacologic impairment of cognitive function. If this is true, then steps should be taken to prevent exposure to lead and to reduce lead levels in individuals of any age, not just young children.
